# Dissecting the exercise pressor reflex in heart failure: A multi-step failure^☆^

**DOI:** 10.1016/j.autneu.2025.103269

**Published:** 2025-03-13

**Authors:** Danilo Iannetta, Fabio Giuseppe Laginestra, D. Walter Wray, Markus Amann

**Affiliations:** a Department of Anesthesiology, University of Utah, Salt Lake City, UT, United States of America; b Department of Clinical and Experimental Sciences, University of Brescia, Brescia, Italy; c Department of Internal Medicine, University of Utah, Salt Lake City, UT, United States of America; d Geriatric Research, Education, and Clinical Center, VA Medical Center, Salt Lake City, UT, United States of America

**Keywords:** Group III/IV muscle afferents, Autonomic nervous system, Sympathetic activity, Blood flow

## Abstract

The contribution of neural feedback originating from exercising limb muscles to the cardiovascular response to exercise was first recognized nearly 100 years ago. Today, it is well established that this influence is initiated by the activation of group III and IV sensory neurons with terminal endings located within contracting skeletal muscle. During exercise, these sensory neurons project feedback related to intramuscular mechanical and metabolic perturbations to medullary neural circuits which reflexively evoke decreases in parasympathetic and increases in sympathetic nervous system activity with the purpose of optimizing central and peripheral hemodynamics. Considerable evidence from animal and human studies suggests that the function of this regulatory control system, known as the exercise pressor reflex (EPR), is abnormal in heart failure and exaggerates sympatho-excitation which impairs the hemodynamic response to exercise and contributes to the functional limitations characterizing these patients. This review briefly introduces the key determinants of EPR control in health and covers the impact of heart failure on the integrity of each of its components and overall function. These include the sensitivity of group III/IV muscle afferents, afferent signal transmission in the spinal cord, and the central integration and processing of sensory feedback within the brainstem. Importantly, although most data relevant for this review come from studies in HFrEF, the limited HFpEF-specific insights are included when available. While arguably not part of the EPR, we also discuss the impact of heart failure on the exercise-induced increase of intramuscular stimuli of group III/IV muscle afferents and end-organ responsiveness to sympathetic/neurochemical stimulation.

## 1. Introduction

Exercise engages a series of neurocirculatory control mechanisms which, in combination with regional vasodilation (Gliemann et al., 2019), facilitate blood flow to meet the increased metabolic demand of the working muscles, while simultaneously assuring adequate perfusion of vital organs (e.g., brain) (Joyner and Dempsey, 2018; Rowell, 1988). Specifically, starting with the onset of exercise, neural “feedforward” and “feedback” mechanisms provide input that is integrated within the nuclei of the medulla oblongata to orchestrate the requisite adjustment of both the parasympathetic and the sympathetic branch of the autonomic nervous system (Wan et al., 2023). The primary feedforward mechanism is generally referred to as “central command” (Goodwin et al., 1972) and includes descending neural signals from higher brain areas that cause parallel activation of cortical motor control regions and neural medullary circuits that regulate the autonomic nervous system (Waldrop et al., 2011). The feedback mechanisms include the arterial and cardiopulmonary baroreflex (Fadel and Raven, 2012), the arterial chemoreflex (Kumar and Prabhakar, 2012) and the exercise pressor reflex (EPR) (Kaufman, 2012; Secher and Amann, 2012). While baroreflex engagement increases parasympathetic activity and reduces sympathetic outflow, central command, the arterial chemoreflex, and the EPR all serve to decrease parasympathetic activity and promote sympatho-excitation (Fisher et al., 2015; Wan et al., 2023). A delicate balance of these mechanisms and their effects on the autonomic nervous system is critical to the maintenance of cardiovascular homeostasis during physical activity.

This balance is impaired in patients with heart failure (HF) (Floras and Ponikowski, 2015). The net effect is an excessive sympathetic drive that debilitates cardiac, renal, and vascular function, limits blood flow to active muscle (Amann et al., 2014) and, as a consequence, impairs neuromuscular fatigue resistance (Weavil et al., 2021) and facilitates exercise intolerance (Schindler et al., 2019). While disease-related abnormalities in various neurocirculatory control mechanisms have been identified to contribute to these impairments (Bunsawat et al., 2023b; Manabe et al., 2023; Toledo et al., 2017), an abnormal EPR function is increasingly recognized to play a major role (Angius and Crisafulli, 2020; Garry, 2011; Grotle et al., 2020; O’Leary and Mannozzi, 2024).

With a specific focus on its afferent arm, this review briefly introduces how the EPR operates in health and then covers both animal and human studies reflecting the impact of HF on the function of this neural mechanism and the integrity of each of its components. These include group III/IV muscle afferents, the transmission of afferent signals within the spinal cord, and the processing of sensory feedback within the medulla. While arguably not part of the EPR, we will also discuss the impact of HF on both the exercise-induced increase of intramuscular stimuli known to activate the afferent arm of the EPR and the responsiveness of the peripheral vasculature to sympathetic stimulation.

Finally, HF patients with a left ventricular ejection fraction ≤40 % are typically classified as HF with reduced ejection fraction (HFrEF), while those with a normal ejection fraction (55–70 %) are classified as HF with preserved ejection fraction (HFpEF) (Schindler et al., 2019). Although the pathophysiology, treatment, and prognosis of these two forms of HF are distinct (Bloom et al., 2017; Borlaug and Borlaug, 2014), both feature abnormal EPR function (Amann et al., 2014; Bunsawat et al., 2023a; Piepoli et al., 2008; Smith et al., 2020). Although most findings related to the impact of HF on EPR function come from animal and human studies in HFrEF, our writing also includes discussions on the limited data available in HFpEF.

## 2. Group III/IV muscle afferents

The afferent arm of the EPR consists of group III and IV muscle afferents, i.e., two types of sensory neurons with their receptive fields located in close proximity to connective tissue and blood vessels, and within the interstitial space of skeletal muscle. Group III muscle afferents are thinly myelinated neurons with mechano-gated channels (e.g., Piezo) (Copp et al., 2016; Ducrocq et al., 2024; Fukazawa et al., 2023) at the terminal end that are sensitive to mechanical deformation. Group IV muscle afferents are unmyelinated neurons with channels (e.g., ASICs) and receptors (e.g., bradykinin and purinergic) that are activated by contraction-induced changes in the concentration of various metabolites within the muscle interstitium (Hanna and Kaufman, 2003; McCord et al., 2009). Although the exact concentration and combination of metabolites required to activate these metabosensitive neurons remain elusive, lactate, ATP, hydrogen ions, potassium, bradykinin, phosphates, and prostaglandins have been suggested to play a key role (Ducrocq and Kaufman, 2020; Light et al., 2008; Pollak et al., 2014; Rotto et al., 1989; Stebbins et al., 1986). Importantly, two sub-categories of metabosensitive neurons have recently been identified. One category includes neurons primarily activated by interstitial metabolite concentrations typical of regular aerobic exercise, while the other category, sometimes referred to as “metabo-nociceptors”, includes neurons activated by noxious metabolite concentrations only seen during ischemic muscle contractions (Jankowski et al., 2013; Light et al., 2008).

It should also be emphasized that the functional distinction between group III and group IV muscle afferents is not absolute, as some fibers in either group are considered polymodal, sensing both mechanical and metabolic stimuli (Adreani et al., 1997; Adreani and Kaufman, 1998). Furthermore, the sensitivity of some mechanosensitive muscle afferents can be influenced by the metabolic milieu of the muscle, as shown by both animal (Adreani and Kaufman, 1998; Hanna and Kaufman, 2003; Kaufman and Rybicki, 1987; Mense and Stahnke, 1983; Rotto et al., 1990; Sinoway et al., 1993) and human (Cui et al., 2007) studies. However, the full extent of this interaction remains unknown.

## 3. EPR function in health

**Fig. 1.**
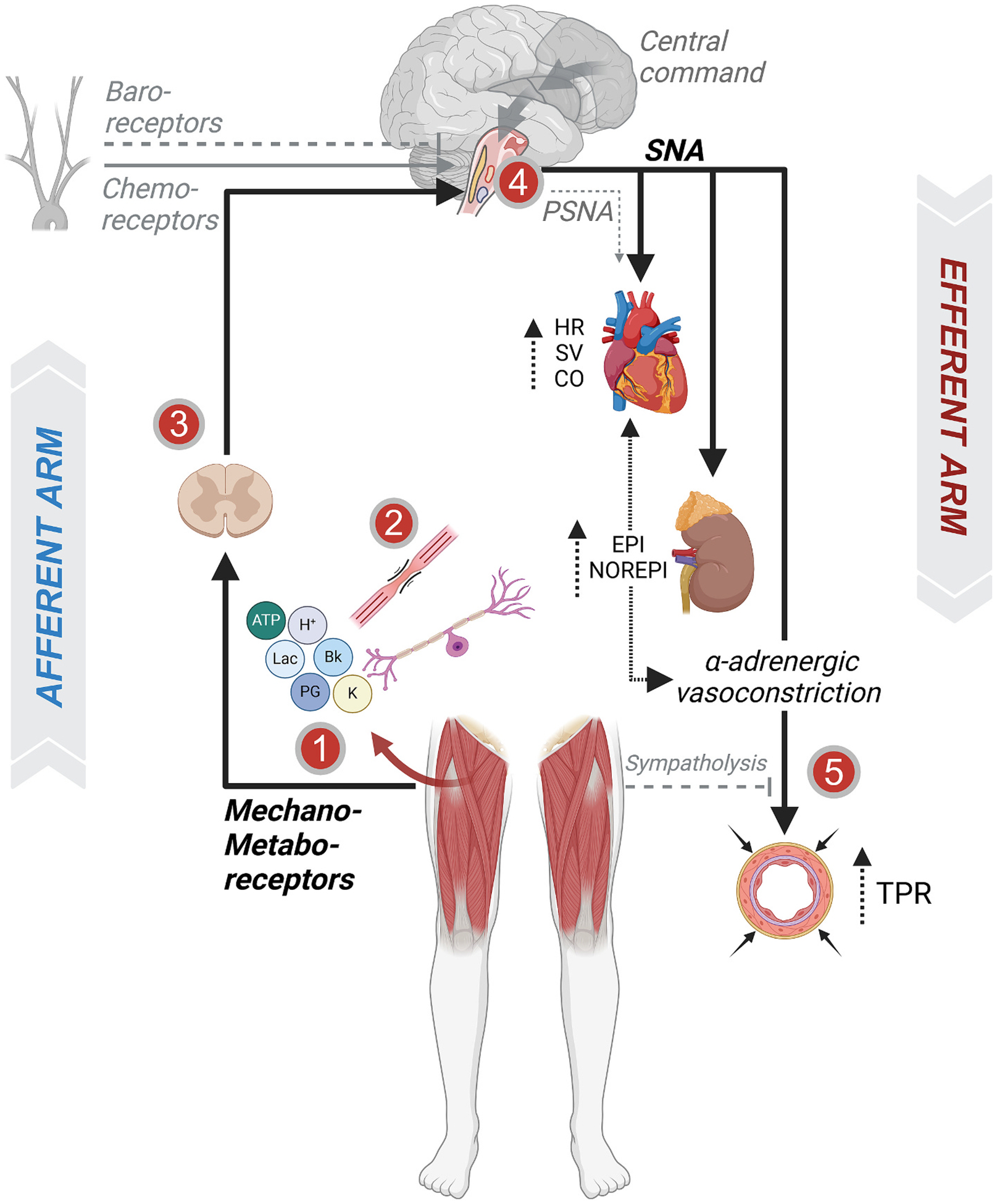
Schematic representation of the exercise pressor reflex (EPR). The left and the right segments of the schematic depict the afferent and efferent arms of the EPR, respectively. Other sympatho-excitatory (i.e., central command and chemoreceptors) and sympatho-inhibitory (baroreceptors) signals are also represented. Numbers denote known sites of EPR-related signaling alterations in heart failure (HF). **(1)** Mechanical and metabolic perturbations during exercise are sensed by the group III/IV muscle afferents. Resulting from disease-related skeletal muscle abnormalities, exercise-induced metabolic perturbation in the muscle interstitium is, at any given workload, larger in patients with HF compared to healthy controls. **(2)** However, the exacerbated metabolic milieu in HF may not necessarily result in overactivation of the metabosensitive afferents and, thus, the EPR. In fact, the sensitivity of group IV muscle afferents has been found to be unchanged or even blunted, and hyper-sensitive group III neurons might largely account for the excessive EPR in HF. **(3)** The dorsal horn is the first level of group III/IV muscle afferent feedback processing within the spinal cord and represents a major site of feedback amplification in HF. Animal studies suggested that group III/IV muscle afferents release more excitatory neurotransmitters in response to muscle contraction and post-synaptic neurons seem to be more sensitive to any given amount of these substances. **(4)** Post-synaptic neurons converge within the cardiovascular control centers of the medulla. In HF, the medullary integration of all incoming neuronal signals may be disrupted by a misbalance between excitatory and inhibitory neurotransmitters. The result of these alterations is an excessive sympathetic outflow which, **(5)** due to a reduced sympatholytic and vasodilatory capacity of the peripheral vasculature, accentuate the increase in total peripheral resistance (TPR) which restricts blood flow to active muscle. ATP: adenosine triphosphate; H^+^: hydrogen ion; Lac: lactate; Bk: bradykinin; K^+^: potassium; PG: prostaglandin; SNA: sympathetic nerve activity; PSNA: parasympathetic nerve activity; HR: heart rate; SV: stroke volume; CO: cardiac output; EPI: epinephrine; NOREPI: norepinephrine; TPR: total peripheral resistance.

The EPR entails the activation of group III/IV muscle afferents which stimulate neural circuits in the brainstem that, in response (i.e., reflexively), alter autonomic nervous system activity with the goal of adjusting cardiac output and vascular tone to optimize perfusion pressure and blood flow, and thus meet the metabolic demand of the exercising skeletal muscle. More specifically, the EPR is triggered by exercise-induced intramuscular mechanical and metabolic stimuli which stimulate receptors and channels located on group III and IV muscle afferents (see above). Once activated, these sensory neurons synapse in the dorsal horn of the spinal cord and project to the medulla oblongata where they connect with neurons of the nucleus of tractus solitarius (NTS), the paraventricular nucleus (PVN), and the ventrolateral medulla (CVLM) (Craig, 2013; Sato and Schmidt, 1973), i.e. brain structures regulating the autonomic nervous system. While isolated activation of the EPR facilitates parasympathetic withdrawal and sympathetic outflow to raise cardiac output and peripheral vasoconstriction (Mitchell et al., 1983; Rowell and O’Leary, 1990), it is important to emphasize that the ultimate adjustment of the autonomic nervous system to exercise is, as recently discussed by Wan and colleagues (Wan et al., 2023), an integrated response and the consequence of various neurocirculatory control mechanisms operating in combination and interacting with each other. A discussion of the impact of HF on these interactions is beyond the scope of this review. Regardless, the hemodynamic consequence of the EPR (and all other neurocirculatory control mechanisms) ultimately depends on the end-organ response (e.g., heart, vasculature) to sympathetic stimulation (Mueller et al., 2017) (Fig. 1).

While the EPR has been shown both to facilitate blood flow to working skeletal muscle and to protect against detrimental decreases in blood pressure by preventing the vasodilatory capacity of exercising limb muscle from outstripping the pumping capacity of the heart (Joyner and Casey, 2015), its primary purpose remains a subject of debate (Joyner, 1991; Joyner, 2006; O’Leary, 2006; Rowell, 1988; Rowell, 1997). Studies in exercising dogs using partial restriction of limb blood flow (via graded inflation of a vascular occluder implanted on the terminal aorta) found that the evoked facilitation of the EPR increased cardiac output and restored ~50 % of the normal hindlimb blood flow (O’Leary and Sheriff, 1995; O’Leary et al., 1999). These animal studies support the idea that the EPR is primarily a blood flow-raising mechanism with the main purpose of limiting a mismatch between muscle O_2_ supply and demand.

Findings in humans are less definitive and suggest that the primary purpose of the EPR may depend on the amount of muscle mass engaged in the exercise. Valuable insight on the role of the EPR in regulating the cardiovascular response to exercise in humans comes from studies using lumbar intrathecal fentanyl, a μ-opioid receptor agonist which attenuates group III/IV-mediated feedback from exercising limb muscles to the brainstem. This approach was recently used to attenuate the EPR during single-leg knee-extension (recruiting a small muscle mass) (Amann et al., 2011; Sidhu et al., 2015) and locomotor (cycling; recruiting a large muscle mass) (Thurston et al., 2023) exercise in healthy individuals. A comparison of these studies revealed muscle-mass dependent differences of the role of the EPR in determining the cardiovascular response to exercise. Specifically, the EPR was found to account for a larger proportion of the blood pressure response, but a smaller portion of the cardiac output response, to exercise recruiting a large compared to a small muscle mass. Furthermore, while the EPR limited leg vascular conductance during locomotor exercise, it enhanced vascular conductance during single-leg knee-extension exercise (i.e., small muscle mass). Finally, the EPR had no effect on leg blood flow during locomotor exercise, but facilitated leg blood flow during single-leg knee-extension exercise (Thurston et al., 2023). These observations suggest that the primary role of the EPR is that of a flow-raising mechanism during small muscle mass exercise, while during locomotor exercise, it may primarily be considered a pressure-raising mechanism that serves the entire body, but without affecting blood flow to exercising skeletal muscle.

## 4. Impact of heart failure on EPR function

The balance between sympatho-excitatory, sympatho-inhibitory, and sympatholytic mechanisms, which ensures optimal hemodynamic responses to exercise in health, is disrupted in HF (Fig. 1). In an animal model of HFrEF, EPR dysfunction appears to contribute to the excessive sympathetically-mediated vasoconstriction that limits limb blood flow (Kaur et al., 2018) and compromises ventricular contractility (Coutsos et al., 2013) during exercise. Initial human evidence for EPR-mediated impairments in the hemodynamic response to voluntary exercise in HF was provided by a study using lumbar intrathecal fentanyl to block group III/IV leg muscle afferent feedback during single-leg knee extension exercise in patients with HFrEF (Amann et al., 2014). While the pharmacological attenuation of the EPR decreased cardiac output and reduced perfusion pressure, it prevented the excessive sympatho-excitation (i.e., reduced norepinephrine spillover), substantially increased leg vascular conductance and blood flow, and reduced end-exercise neuromuscular fatigue in all individuals with HFrEF (Amann et al., 2014). These findings suggest that the EPR facilitates the central hemodynamic response to exercise in HFrEF patients, but also exaggerates sympathetic vasoconstriction and impairs limb blood flow which contributes to the exercise intolerance characterizing this population (Amann et al., 2014). Later studies (Smith et al., 2020) using the same pharmacological approach in HFrEF patients performing bicycle exercise confirmed these initial findings. However, it was also documented that, in contrast to knee-extensor exercise (Amann et al., 2014), the EPR constrains stroke volume and cardiac output during locomotor exercise in HFrEF, presumably due to a larger EPR-mediated increase in cardiac afterload (Smith et al., 2020).

The following sections discuss the impact of HF on the integrity of the components involved in the EPR which, individually or in conjunction, determine its consequence for the autonomic nervous system response to exercise (Fig. 1). Of note, as the hemodynamic response to EPR activation is also affected by the magnitude of the exercise-induced increase of intramuscular stimuli for group III/IV muscle afferents and end-organ responsiveness to sympathetic stimulation, we include an overview covering the impact of HF on those determinants.

## 5. Impact of HF on the sensitivity and activation of the EPR

HF considerably alters the morphology of skeletal muscle (Lunde et al., 2001; Paneroni et al., 2018). Animal studies offer evidence for muscle atrophy (Bowen et al., 2015; Simonini et al., 1996) and a progressive shift in fiber type distribution, directionally from slow- to fast-twitch fibers in rat models of both HFrEF (Knapp et al., 2020; Sabbah et al., 1993; Vescovo et al., 1998) and HFpEF (Saw et al., 2022). Furthermore, independent of fiber type expression, muscle oxidative capacity has been found to be compromised in rats with HFrEF (Garnier et al., 2003) and HFpEF (Bowen et al., 2015; Espino-Gonzalez et al., 2021), and this impact is thought to be mediated by disease-related impairments in muscle O_2_ delivery and mitochondrial efficiency (Poole et al., 2018). These structural and functional alterations, which appear to be accentuated in animal models of HFrEF compared to HFpEF (Seiler et al., 2016), are independent of muscle disuse and low activity levels (Delp and O’Leary, 2004; Simonini et al., 1996), two HF-specific characteristics that can independently accentuate these muscle alterations in humans (Nunes et al., 2022). Investigations in patients with HF corroborate these animal-based findings (Adams et al., 2017; Kumar et al., 2019), documenting muscle atrophy, fiber-type shifting, capillary rarefaction, and mitochondrial dysfunction in HFrEF (Drexler et al., 1992; Mancini et al., 1989) and HFpEF (Goto et al., 2021; Kitzman et al., 2014; Molina et al., 2016; Zamani et al., 2021). Interestingly, in contrast to what has been reported in animals (Seiler et al., 2016), skeletal muscle dysfunction appear to be more pronounced in patients with HFpEF compared to HFrEF (Bekfani et al., 2020).

These HF-related muscle alterations result in a greater engagement of fast-twitch muscle fibers and a greater reliance on anaerobic energy sources during exercise at any given workload, which leads to larger intramuscular metabolic perturbation compared to controls (Mancini et al., 1988; Massie et al., 1987; Melenovsky et al., 2018; Wilson et al., 1985). Considering that the magnitude of the EPR increases with increases in contraction-induced metabolic perturbation (Petrofsky et al., 1980; Petrofsky et al., 1981; Wilson et al., 1995; Xing et al., 2008), it is reasonable to speculate that at least a part of the exaggerated EPR in HF may result from the excessive exercise-induced intramuscular metabolite accumulation and the associated stronger activation of metabosensitive muscle afferents and, therefore, the metabolic component of the EPR.

Despite this evidence, findings related to the impact of HF on metaboreflex function are discordant. For example, the cardiovascular response to the activation of metabosensitive group IV muscle afferents via various chemical stimuli has been suggested to be unchanged (Butenas et al., 2022) or even blunted (Smith et al., 2005b; Smith et al., 2005c; Wang et al., 2010) in animal models of HFrEF. Human HFrEF studies designed to isolate the metaboreflex component of the EPR are also divergent. As a sidenote, most of the studies focusing on the metaboreflex in humans are based on exercise recruiting a small muscle mass and post-exercise circulatory occlusion (PECO) to trap exercise-induced intramuscular metabolites in an attempt to isolate the contribution of the muscle metaboreflex to the overall EPR response (Alam and Smirk, 1937). Some investigations based on this approach found the metaboreflex component of the EPR to be exaggerated (Notarius et al., 2001; Piepoli et al., 1996), while others observed normal (Carrington et al., 2001) or even blunted metaboreflex function (Sterns et al., 1991). Interestingly, other studies showed that the cardiovascular effect evoked by the metaboreflex component may progressively decrease with increases in the severity of HFrEF (Kon et al., 2004; Negrão et al., 2001). PECO studies in HFpEF patients are similarly conflicting, with some documenting a normal (Sarma et al., 2021) and others a blunted (Bunsawat et al., 2023a) hemodynamic response to the activation of the metaboreflex component of the EPR. Thus, despite considerable skeletal muscle abnormalities (see above), the resulting greater intramuscular stimulation of metabosensitive muscle afferents might not necessarily exaggerate the EPR in either HF phenotype. It is important to consider, however, that both the magnitude (Estrada et al., 2020; Savard et al., 1989) and the function (Thurston et al., 2023) of the EPR are sensitive to the muscle mass engaged in the exercise and the interaction between the mechanoreflex and the metaboreflex component of the EPR (Bell and White, 2005; Cui et al., 2008); thus, the relevance of PECO-based findings for EPR function during locomotor exercise remains unclear.

**Fig. 2.**
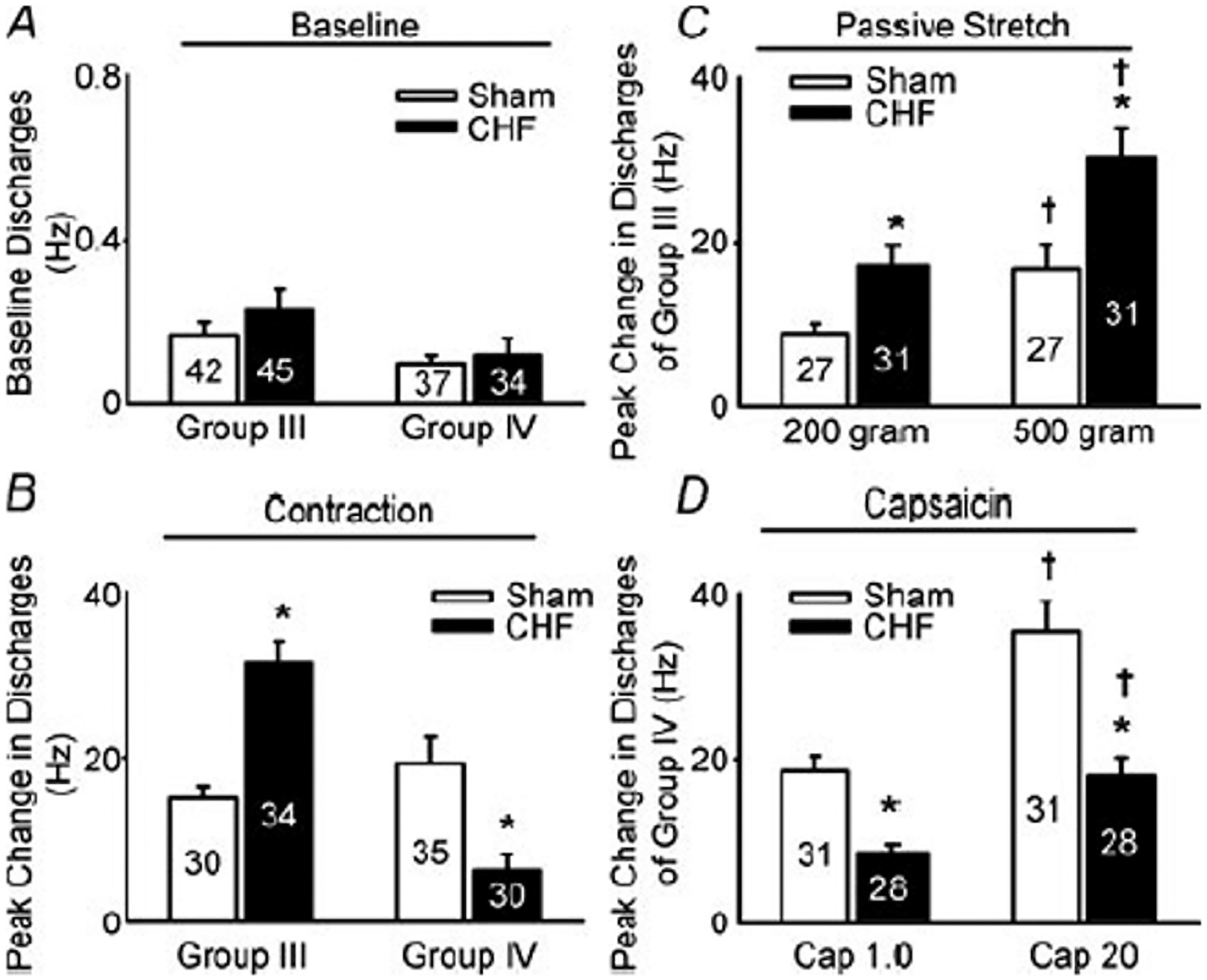
Discharge frequency of group III and IV afferents in response to static contraction induced by electrical stimulation of L_5_ ventral root, to passive stretch and to capsaicin in sham and rats with heart failure (CHF). Mean data illustrating **(A)** the baseline discharge of group III and IV muscle afferents in sham and HF rats, and **(B)** the responses of these sensory neurons to static contraction induced by electrical stimulation of L_5_ ventral root. Panel C and D illustrate mean data showing the response of group III and IV muscle afferents to **(C)** two levels of passive stretch and **(D)** two doses of capsaicin in sham and CHF rats. Data are expressed as mean ± SEM. *P < 0.05 vs. sham, †*P* < 0.05 vs. lower level of stretch or lower dose of capsaicin. From Wang et al.(Wang et al., 2010).

Although factors contributing to the potential blunting of the metaboreflex component of the EPR in HF are unknown, reductions in the sensitivity of metabosensitive group IV muscle afferents have been suggested. Support for this idea comes from a rat study recording the discharge of group IV muscle afferents in response to intramuscular injection of capsaicin, which selectively activates transient receptor vanilloid 1 (TRPV1) metaboreceptors on group IV neurons (Wang et al., 2010). These experiments showed that the neural response to this chemical stimulus is, compared to controls, lower in rats with HFrEF, suggesting a blunted responsiveness of metabosensitive muscle afferents (Fig. 2). This is in agreement with earlier work documenting diminished expression of mRNA for the capsaicin receptor TRPv1 within dorsal root ganglions of the sensory neurons innervating skeletal muscle of HFrEF rats (Smith et al., 2005c). While the reasons for the blunted sensitivity and the lower expression of metaboreceptors in HF remain unclear, the repeated exposure to abnormally high metabolite concentrations has been suggested to contribute to these disease-related alterations (Liu et al., 2010; Sanz-Salvador et al., 2012; Smith et al., 2005c).

So, why is the EPR exaggerated in HF despite a potentially blunted metaboreflex component? Animal studies suggest that the abnormal response is primarily determined by an overactivity of the mechanoreflex component of the EPR (Butenas et al., 2024; Koba et al., 2008; Li et al., 2004; Middlekauff et al., 2001; Smith et al., 2005b). Human studies focusing on the impact of HF on the mechanoreflex are surprisingly limited. Findings in support of this concept come from a study utilizing passive wrist flexion–extension exercise in HFrEF patients. While this maneuver increased muscle sympathetic nerve activity in the patients, it had no effect in healthy controls, suggesting that HFrEF is associated with an overactive mechanoreflex (Middlekauff et al., 2004). To our knowledge, no studies to date have characterized muscle mechanoreflex function in patients with HFpEF.

Different mechanisms have been proposed to intensify the mechanoreflex in HF. Wang and colleagues recorded the discharge of group III muscle afferents in response to a mechanical stimulus and found that muscle stretch increases the firing rate of these sensory neurons more in a rat model of HFrEF than in controls (Wang et al., 2010) (Fig. 2). While the increased responsiveness of group III afferents likely contributes to the abnormal EPR in HF, the exact causes underlying this functional change remain unclear. One potential explanation is a (over-)compensatory response to the blunted sensitivity of group IV muscle afferents (Garry, 2011). This hypothesis is indirectly supported by the observation that capsaicin-mediated destruction of TRPV1 receptors on group IV muscle afferents in healthy newborn rats leads to the development of an exaggerated mechanoreflex response to muscle stretch (Smith et al., 2005c). Another potential explanation for the heightened responsiveness of group III neurons in HF is a disease-related functional change of receptors on group III/IV afferents or an increase in the expression of mechanosensitive receptors. For instance, ASIC1a channels, which only respond to metabolic perturbation in healthy animals (Kaufman, 2012), were found to be sensitive to both metabolic and mechanical stimuli in a rat model of HFrEF (Butenas et al., 2022). Furthermore, the expression of the ATP-sensitive P2X receptors, which can indirectly be activated by a mechanical stimulus causing Piezo channels to release ATP into the muscle interstitium (Wei et al., 2019), is greater in the dorsal root ganglion of HFrEF rats than in controls (Gao et al., 2007). Similarly, the dorsal root ganglion expression of bradykinin B2 receptors, which, upon activation, amplify P2X-mediated pressor responses, is greater in HFrEF compared to control rats (Xing and Li, 2016). Important in this context is the observation that pharmacological blockade of P2X receptors reduces the response of group III muscle afferents to muscle stretch to a greater extent in HF vs controls (Wang et al., 2010).

Another potential contributor to the exaggeration of the mechanoreflex component of the EPR in HF is the fact that increased levels of metabolites within the interstitium of contracting skeletal muscle (as is the case in HF, see above) can sensitize group III muscle afferents (Li et al., 2004; Li and Sinoway, 2002; Sinoway et al., 1993). Other studies suggest that the cyclooxygenase (COX) pathway, which leads to the formation of prostaglandins in the working muscle, is altered in animal models of HFrEF and that this may contribute to the sensitization of mechanoreceptors (Morales et al., 2012). However, definitive evidence for a considerable role of COX-2 in human HF is missing. Indirect support for its significance in HFrEF is provided by the observation that the lower muscle sympathetic nerve activity during passive leg movement after, compared to before, a 4-month exercise program is associated with a lower muscle COX-2 expression (Antunes-Correa et al., 2014).

Taken together, the majority of human and animal studies suggest that disease-related alterations in the expression and sensitivity of receptors and channels on group III/IV muscle afferents contribute to the dysfunctional EPR in HF. However, it remains unclear whether abnormalities in the mechanoreflex, the metaboreflex, or both components of the EPR ultimately determine the disease-related impact on this cardiovascular reflex. While most animal studies suggest that the metaboreflex component of the EPR may not be considered as hyper-active in HFrEF or HFpEF and that the mechanoreflex component is, at least in HFrEF, overactive, findings in humans remain unclear. More patient studies are needed to clarify the impact of HF on the two components of the EPR in humans.

## 6. Impact of HF on spinal transmission of group III/IV muscle afferent feedback

**Fig. 3.**
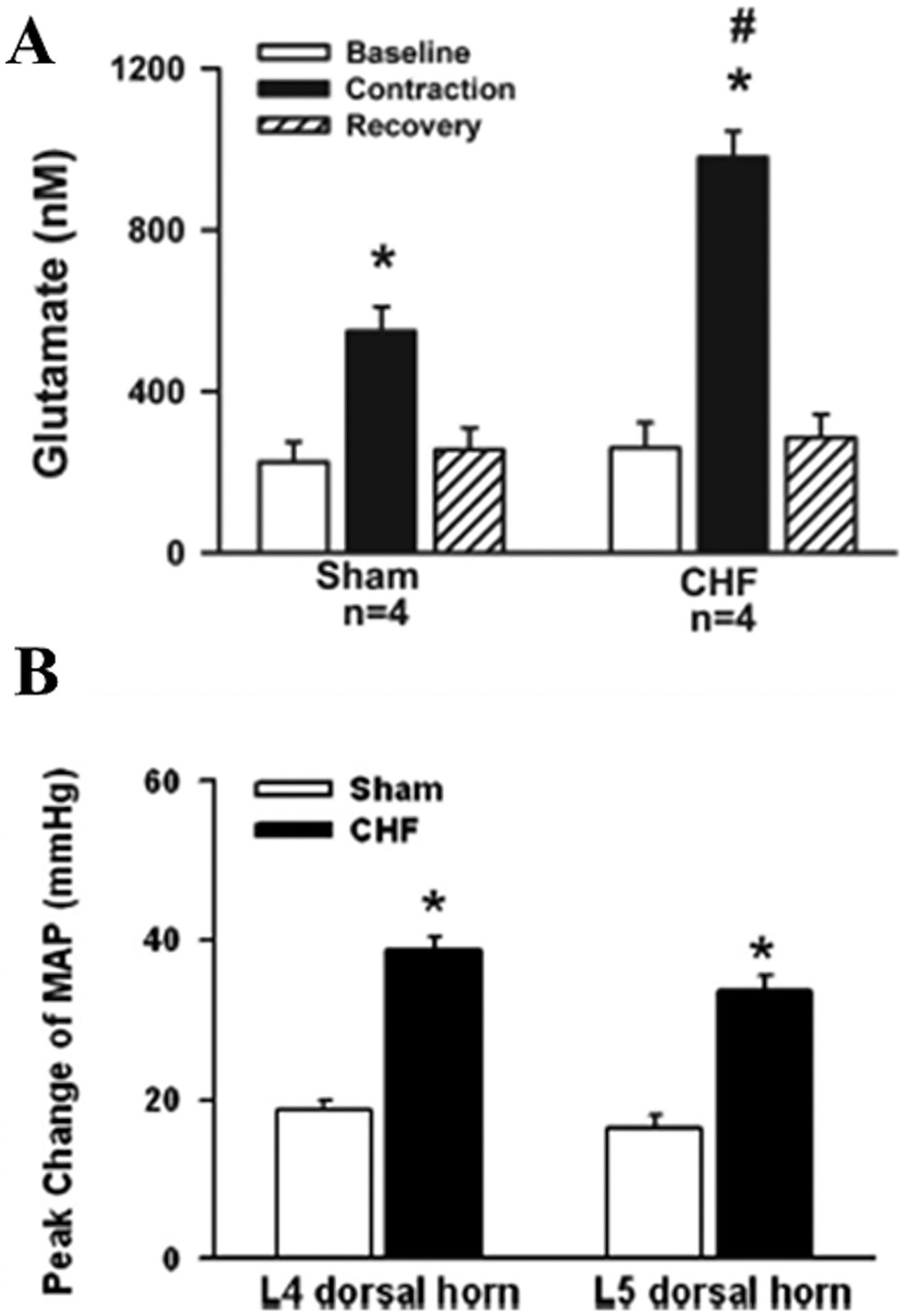
Impact of HF on afferent signal transmission within the dorsal horn of the spinal cord. Panel **(A)**: microdialysis data reflecting increased glutamate release during a 4-min intermittent static contraction (30-s on, 30-s off) in congestive heart failure (CHF) rats compared with sham-operated (sham) rats. Data are expressed as mean ± SEM. * *P* < 0.05 vs. baseline; # *P* < 0.05 vs. sham rats. Panel **(B)**: effects of microinjection of glutamate (10 mM, 100 nl) into the L_4_ or L_5_ dorsal horn on mean arterial pressure (MAP) in sham and CHF rats. ABP, arterial blood pressure. Data are expressed as mean ± SE; *n* = 6 rats/group. *P < 0.05 vs. sham rats. Adapted from Wang et al.(Wang et al., 2015).

A number of neurotransmitters and receptors are involved in the synaptic transmissions of group III/IV muscle afferent signals to neurons within the dorsal horn of the spinal cord. Amongst these, excitatory amino acids are thought to play a primary role (Adreani et al., 1996; Hand et al., 1996) and have been demonstrated to represent a major site of signal dysregulation, at least in HFrEF. Using in vivo microdialysis, Wang and colleagues demonstrated that electrically-induced contractions of the triceps surae evoke a larger release of glutamate within the dorsal horn of HFrEF rats compared to controls (Wang et al., 2015). Importantly, to account for any potential difference in the magnitude of glutamate release, they also performed controlled exogenous microinjections of this neurotransmitter within the same pool of neurons and found the subsequent increase in blood pressure to be greater in the HFrEF rats compared to controls. Predictably, in response to injection of a glutamate receptor antagonists, the reduction in blood pressure was greater in the HFrEF rats compared to controls (Fig. 3). These findings demonstrate that group III/IV muscle afferent feedback elicits a greater release of glutamate in the dorsal horn of HFrEF rats, and that post-synaptic dorsal horn neurons are more sensitive to a given amount of this neurotransmitter (Wang et al., 2015). The spinal cord is therefore a potential locus of considerable amplification of signals from group III/IV muscle afferent neurons and, thus, the exaggerated EPR in HFrEF. There is currently no data on the impact of HFpEF on the spinal transmission of feedback from these muscle afferents. It is worth mentioning that other mechanisms modulating afferent signal transmission at the level of the spinal cord involve nitric oxide (NO) (Li and Mitchell, 2002; Wilson et al., 1999) and angiotensin (Stebbins and Bonigut, 1995). However, the impact of HF on the integrity of these pathways is unknown.

## 7. Impact of HF on the central integration of group III/IV muscle afferent feedback

Incoming neural signals from central command, arterial baroreceptors, arterial chemoreceptors, and group III/IV muscle afferents are integrated within the NTS, CVLM, and PVN of the brainstem (Mueller et al., 2017). This integration involves several neurotransmitters including sympatho-inhibitory NO and γ-aminobutyric acid (GABA), and sympatho-excitatory glutamate and angiotensin II (Li and Patel, 2003). The availability and balance of these neurotransmitters within the brain influence the integration of incoming signals and the magnitude of the resulting sympathetic outflow; importantly, HF can alter this balance (Kleiber et al., 2008; Zheng et al., 2005).

One potential HF-related alteration of the brain integration of group III/IV muscle afferents relates to the expression and availability of NO. Cerebral NO buffers incoming sympatho-excitatory signals opposing to the glutamatergic system and is particularly abundant in the NTS and PVN (Vincent and Kimura, 1992). Studies in barodenervated healthy cats have shown that an increase in the production and availability of NO within the NTS (via injection of the NO substrate L-arginine) attenuates the blood pressure response to electrically-induced static contractions of the triceps surae (Smith et al., 2005a). Importantly, HF can unfavourably alter the equilibrium between NO and glutamate (Li et al., 2003; Li and Patel, 2003), such that NO concentrations in the NTS and PVN of rats with HFrEF is lower than in controls (Hirooka et al., 2003; Patel et al., 1996; Zhang et al., 1998). This has led to the speculation that low levels of NO would predispose neurons of the NTS and the PVN to become more excitable to incoming afferent stimuli leading to the generation of a greater sympathetic outflow and, thus, pressor response. In fact, in animal models of HFrEF gene transferring of neuronal NO into the PVN has been shown to diminish resting sympathetic outflow, likely via inhibiting glutamate receptors (which facilitate sympatho-excitation) (Horn et al., 1994; Zheng et al., 2011). Furthermore, four weeks of exercise training in rats with HFrEF was reported to normalize NO availability within the PVN and that this improvement was associated with blunted renal sympathetic nerve activity (Zheng et al., 2005). Although findings directly linking EPR abnormalities with cerebral NO are currently not available in animal models of HF, studies in hypertensive rats have shown that inhibition of NO synthase in the NTS via injections of L-NAME resulted in a smaller rise in blood pressure in response to passive stretch compared to normotensive controls (Leal et al., 2012). Considering that animal models of hypertension and HF both suffer from excessive sympatho-excitation, this observation indirectly supports the idea that a disease-related reduction in NO within the NTS may play a critical role in amplifying signals from group III/IV muscle afferents and, thus, contribute to the abnormal EPR in HF. Despite these convincing findings in animals, human experimental evidence for a role of altered levels of NO in exaggerating the EPR in HF is scarce and inconclusive (Eggebeen et al., 2016; Hirai et al., 2017; Notay et al., 2017; Young et al., 2009), likely because of the difficulty of isolating its neuronal function. Data in HFpEF are currently not available.

**Fig. 4.**
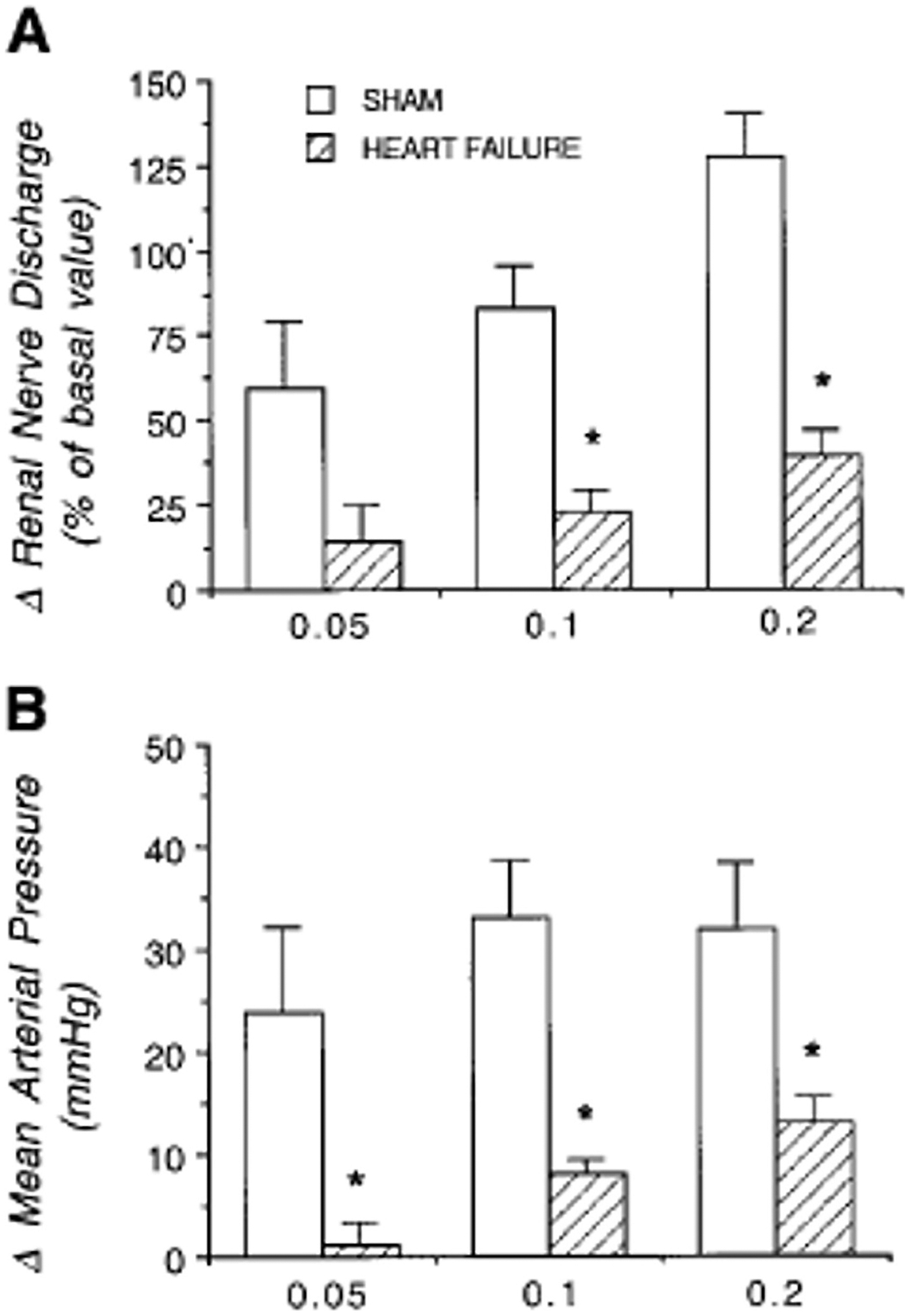
Effect of microinjection of bicuculline into the PVN on **(A)** renal nerve discharge and **(B)** mean arterial blood pressure in sham-operated control and heart failure rats. Values represent means ± SE; * *P* < 0.05 vs. sham. Adapted from Zhang et al.(Zhang et al., 2002).

Another neurotransmitter potentially involved in altering signal integration within the brain is GABA, which is tightly linked to the activity of NO (Li and Patel, 2003). GABA has been demonstrated to influence the regulation of the EPR within both the spinal cord and the PVN. Specifically, injection of bicuculline (a GABA receptor antagonist) into the L_4_/L_5_ dorsal horns of healthy rats increases the EPR (i.e. blood pressure and heart rate) response to electrical stimulation of the ipsi-lateral ventral roots (Wang et al., 2013). Interestingly, in animal model of HFrEF microinjection of bicuculline into the PVN was less effective in lowering renal sympathetic activity compared to controls (Zhang et al., 2002) (Fig. 4). This observation suggested that in HFrEF, post-synaptic GABA receptors in the PVN may be less sensitive to GABAergic stimulation, which results in a reduced ability to buffer sympatho-excitatory signals within the brain. There is currently no data from animals with HFpEF.

Collectively, these findings suggest that the complex molecular mechanisms processing incoming signals (from central command and various feedback mechanisms) and modulating sympathetic outflow within the cardiovascular nuclei of the brain are altered in HFrEF. Overall, it appears that this disruption occurs as a result of an imbalance between the inhibitory effects of NO and GABA, and the excitatory effects of glutamate. Other mechanisms potentially involved in the altered brain integration of incoming group III/IV muscle afferent signals include angiotensin II (Wang et al., 2014; Zucker et al., 2015), oxidative stress (Zucker and Pliquett, 2002), and inflammatory processes (Yu et al., 2007). However, direct evidence for a role of these mechanisms in the brain-mediated exaggeration of the EPR in HF is currently missing.

## 8. Impact of HF on the end-organ responsiveness to sympathetic stimulation

While disease-related changes in the sensing functionality, spinal transmission, and central integration of group III/IV muscle afferents contribute to the excessive sympatho-excitation in HF, the consequence of the elevated sympathetic outflow at the end-organ requires careful consideration. Indeed, vascular conductance and blood flow in the peripheral vasculature are ultimately determined by the balance between sympathetic activity and the ability of the vasculature to dilate (i.e., functional sympatholysis) (Gliemann et al., 2019). While the former depends on overall sympathetic outflow and vascular transduction (which is defined as the system’s efficiency in “translating” a burst of sympathetic activity into the end-organ response (e.g., vasoconstriction)), the latter is mainly governed by endothelial-dependent vasodilatory signals (Young et al., 2021). The modulation of vascular tone is primarily mediated by α-adrenergic receptor function (which includes changes in receptor density and sensitivity), and by the dynamics involving spillover, reuptake, and clearance of neurotransmitters (Fairfax et al., 2013). Chronically heightened sympathetic activity can, over time, lead to a reduction in adrenergic receptor density, expression, and sensitivity. While this is generally accepted for β-adrenergic receptors in HF (Bristow, 1993; Lohse et al., 2003; Mahmood et al., 2022), findings related to α-receptors are less clear. Of note, a reduction in α-adrenergic responsiveness would require a higher level of sympathetic activation to achieve the desired end-organ target response (e.g., vasoconstriction).

Animal studies investigating α-adrenergic control of the vasculature have produced inconsistent results. Examination of isolated vessels from canine HFrEF models have reported both increased (Forster and Armstrong, 1990; Forster et al., 1989) and decreased α-receptor responsiveness (Main et al., 1991). This variability is likely attributable to the specific function of the vascular bed from which the vessels were obtained. Interestingly, Thomas et al. showed that while α-adrenergic responsiveness to sympathetic efferent stimulation was similar between HFrEF rats and controls, the HFrEF animals had a lower sympatholytic capacity that was related to oxidative stress and NO availability (Thomas et al., 2001).

Furthermore, Nardone and colleagues recently reported a blunted transduction of muscle sympathetic nerve activity in resting patients with HFrEF and speculated that this might, at least in part, be due to α_1_ adrenoceptor saturation or desensitization resulting from defective norepinephrine re-uptake (Nardone et al., 2022). Using a pharmacologic approach, Barrett-O’Keefe and colleagues examined alpha adrenergic responsiveness through intra-arterial infusions of phenylephrine (α_1_-agonist) and phentolamine (a non-selective α_1/_α_2_-antagonist) in patients with HFrEF and controls at rest and during single-leg knee extension exercise (Barrett-O’Keefe et al., 2019). At rest, phenylephrine administration provoked a smaller decrease in leg blood flow and vascular conductance in HFrEF compared to controls, indicating reduced α_1_-adrenergic responsiveness. In contrast, complete pharmacologic “sym-pathectomy” achieved via phentolamine infusion increased leg vascular conductance and blood flow more in HFrEF compared to controls at rest and during exercise, suggesting that exaggerated sympathetic restraint limits skeletal muscle blood flow in these patients. Additionally, similar to the findings in animals (Thomas et al., 2001), HFrEF patients were characterized by a decreased magnitude in the exercise-induced “lysis” of sympathetic restraint (Barrett-O’Keefe et al., 2019), an impairment potentially related to the patients’ limited endothelial-dependent vasodilatory capacity (Kubo et al., 1991).

In contrast to these observations, studies utilizing infusions of α_1_ and α_2_-adrenergic agonists (Indolfi et al., 1994), or tyramine (Munch et al., 2018), which facilitate the release of endogenous norepinephrine, found no difference in the drug-induced reductions in blood flow and vascular conductance in patients with HFrEF and controls, suggesting a preserved α-adrenergic responsiveness in the patients. Furthermore, the sympatholytic capacity during dynamic single-leg exercise was found to be preserved in HFrEF patients and even improved after six weeks of exercise training (Munch et al., 2018). While the reasons for the discrepancy between the studies outlined above are not immediately apparent, significant differences in methodology, drug dosing, vascular region, and participant characteristics (e.g. disease timing or age) may explain the divergent findings.

**Fig. 5.**
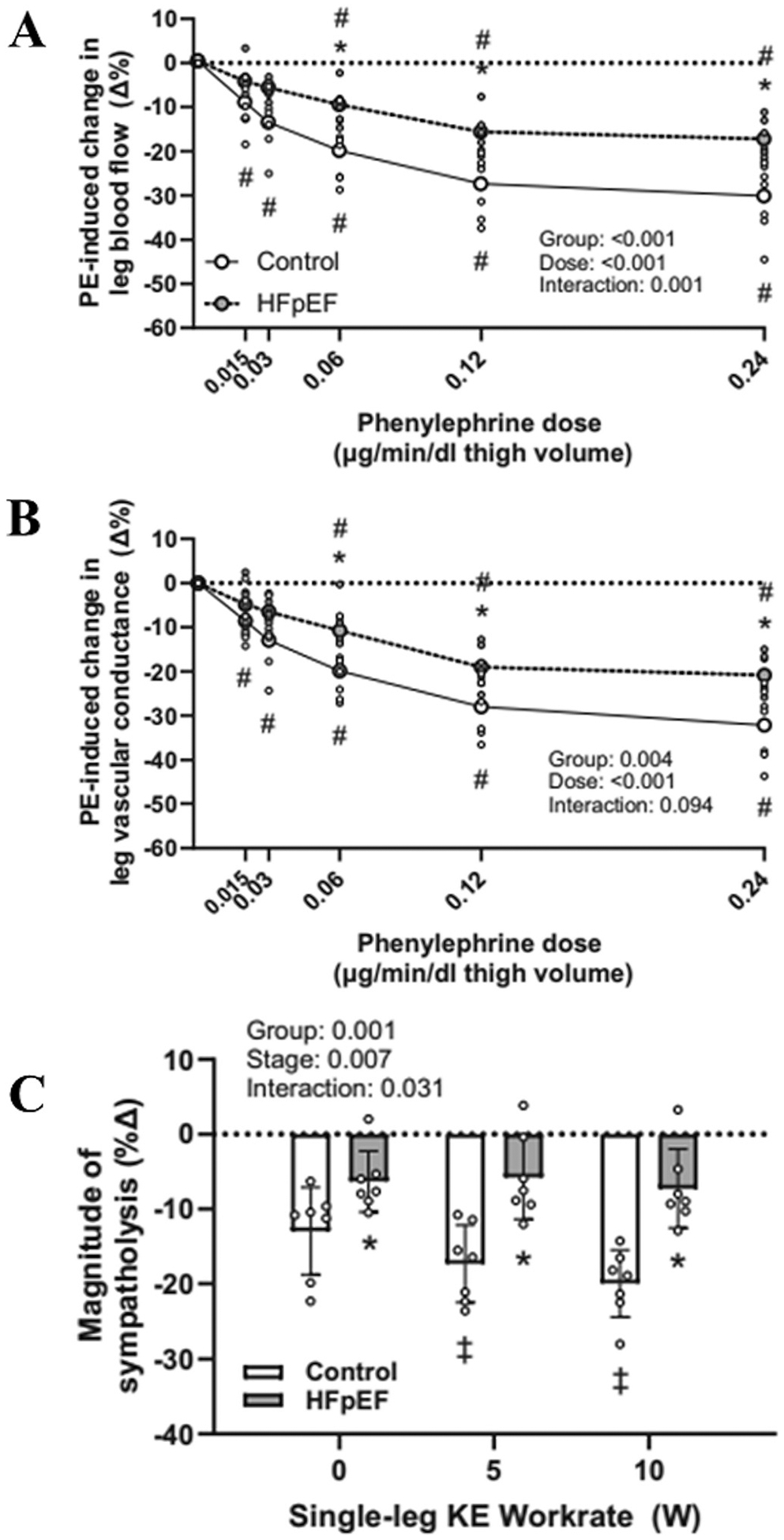
Phenylephrine-induced changes (%) in leg blood flow **(A)** and leg vascular conductance **(B)** at rest, and the magnitude of sympatholysis during single leg knee extension exercise **(C)** in control participants and in heart failure patients with preserved ejection fraction (HFpEF). * *P* < 0.05 vs. control; # *P* < 0.05 vs. pre-infusion. ‡P < 0.05 vs. 0 W. Adapted from Alpenglow et al. (Alpenglow et al., 2024).

Less is known about vascular transduction and α-adrenergic control in HFpEF. A recent study in a large cohort found that sympathetic transduction of muscle sympathetic nerve activity is lower in patients with HFpEF (Takeda et al., 2024) compared to controls. To investigate whether this could be related to α-adrenergic responsiveness and restraint, Alpenglow and colleagues utilized the same pharmacological approach previously used by in HFrEF (Barrett-O’Keefe et al., 2019). Similar to the findings in HFrEF, they observed a lower α-adrenergic responsiveness and excessive sympathetic restraint in the HFpEF patients, which was accompanied by a lower sympatholytic capacity (Alpenglow et al., 2024) (Fig. 5). Interestingly, these observations in HFpEF patients using sympathomimetic drugs support and extend the findings of a previous study utilizing lower body negative pressure to reflexively increase sympathetic nerve activity that demonstrated impaired functional sympatholysis in the vasculature of the upper limbs in this patient group (Alpenglow et al., 2023).

Taken together, evidence addressing the impact of HFrEF on sympathetic transduction, α-adrenergic responsiveness, and sympatholysis remains conflicting, with some studies suggesting normal function (Indolfi et al., 1994; Munch et al., 2018), while others suggest disease-related impairments (Barrett-O’Keefe et al., 2019). In contrast, the limited studies in HFpEF consistently demostrated impairments in sympathetic transduction, α-adrenergic responsiveness, and sympatholysis (Alpenglow et al., 2024; Alpenglow et al., 2023).

## 9. Summary and conclusion

The EPR is exaggerated in HFrEF and likely HFpEF patients. Malfunction in this neurocirculatory control mechanism can result from disease-related alterations of any component, or control site, determining the EPR. Based on current insights, the extent of EPR abnormalities in HF may ultimately depend on the number of dysregulated sites. Pharmacological or physical rehabilitation-based interventions that normalize the function of any of these components/sites will likely improve EPR function and, thus, reduce the symptoms associated with HF. Finally, more HFpEF data are needed to allow for a better differentiation between the two HF phenotypes.

## Data Availability

No data was used for the research described in the article.
